# A non-enteric adenovirus A12 gastroenteritis outbreak in Rio de Janeiro, Brazil

**DOI:** 10.1590/0074-02760160030

**Published:** 2016-06

**Authors:** Silvana Augusta Rodrigues Portes, Eduardo de Mello Volotão, Monica Simões Rocha, Maria Cristina Rebelo, Maria da Penha Trindade Pinheiro Xavier, Rosane Maria de Assis, Tatiana Lundgren Rose, Marize Pereira Miagostovich, José Paulo Gagliardi Leite, Filipe Anibal Carvalho-Costa

**Affiliations:** 1 Fundação Oswaldo Cruz, Instituto Oswaldo Cruz, Laboratório de Virologia Comparada e Ambiental, Rio de Janeiro, RJ, Brasil; 2 Laboratório Central de Saúde Pública, Rio de Janeiro, RJ, Brasil; 3 Fundação Oswaldo Cruz, Escritório Técnico Regional Fiocruz Piauí, Teresina, PI, Brasil

**Keywords:** gastroenteritis, adenovirus, quantitative polymerase chain reaction

## Abstract

A gastroenteritis outbreak that occurred in 2013 in a low-income community in Rio de Janeiro was investigated for the presence of enteric viruses, including species A rotavirus (RVA), norovirus (NoV), astrovirus (HAstV), bocavirus (HBoV), aichivirus (AiV), and adenovirus (HAdV). Five of nine stool samples (83%) from patients were positive for HAdV, and no other enteric viruses were detected. Polymerase chain reaction products were sequenced and subjected to phylogenetic analysis, which revealed four strains and one strain of non-enteric HAdV-A12 and HAdV-F41, respectively. The HAdV-A12 nucleotide sequences shared 100% nucleotide similarity. Viral load was assessed using a TaqMan real-time PCR assay. Stool samples that were positive for HAdV-A12 had high viral loads (mean 1.9 X 10^7^ DNA copies/g stool). All four patients with HAdV-A12 were < 25 months of age and had symptoms of fever and diarrhoea. Evaluation of enteric virus outbreaks allows the characterisation of novel or unique diarrhoea-associated viruses in regions where RVA vaccination is routinely performed.

Acute gastroenteritis (AGE) is a common cause of morbidity and mortality in children ≤ 5 years of age in developing countries. Worldwide, the most common viruses responsible for AGE are rotavirus A (RVA), norovirus (NoV), adenovirus (HAdV) and astrovirus (HAstV) (Corcoran et al. 2014). Recently, newly identified viruses, such as bocavirus (HBoV) and aichivirus (AiV), were associated with AGE; however, further studies are required to demonstrate the relative efficiency of these viruses as aetiologic agents of AGE (Portes et al. 2015).

HAdVs are non-enveloped double stranded DNA viruses belonging to the family *Adenoviridae.* These viruses are associated with several pathologies, including respiratory infections, conjunctivitis and AGE. More than 50 HAdV types exist and are categorised into seven groups (A-G). HAdVs are associated with outbreaks and sporadic cases of AGE in children and adults, especially enteric viruses F40 and F41 (Lee et al. 2012). Non-enteric HAdVs are less frequently associated with AGE (Moyo et al. 2014).

Brazil introduced universal RVA vaccination in 2006, which significantly reduced the prevalence of RVA infection in children with AGE (Carvalho-Costa et al. 2011). Although effective in the prevention of RVA infection, the vaccine does not prevent infection due to other viral or bacterial agents. Furthermore, the RVA vaccine elicits greater protection from infection by Wa-like RVA strains than infection by other strains. Enhanced AGE surveillance is required to identify viral aetiological agents that are less frequently reported, especially with respect to AGE outbreaks. Recent evidence from laboratory - and hospital-based viral AGE surveillance has shown an increased prevalence of NoV, HAstV and HAdV (Ferreira et al. 2012, de Andrade et al. 2014).

In this study, we characterised the viral agents associated with an outbreak of AGE that occurred in Rio de Janeiro, Brazil.

In March of 2013, the Surveillance Epidemiology System was informed of an AGE outbreak that affected 19 individuals in a low-income community in the Bangu neighbourhood of the municipality of Rio de Janeiro, in the state of Rio de Janeiro, Brazil. The outbreak was isolated, affecting people living in close proximity, and lasted less than 10 days. Ten faecal samples were collected from nine patients (six male and three female) who were receiving health care in a local public medical centre (Table). Health authorities from Rio de Janeiro collected single faecal samples on the third or fourth day following the onset of symptoms, except for one patient, from whom two samples were collected. The faecal samples were then sent to the Rotavirus Regional Reference Laboratory/Laboratory of Comparative and Environmental Virology (LRRR/LVCA), FIOCRUZ, for analysis for enteric viruses. All samples were negative for bacterial and protozoal agents, as determined previously by the Central Public Health Laboratory (LACEN). This study is part of a larger project, which was approved by the Ethical Research Committee of the Oswaldo Cruz Foundation (FIOCRUZ) (311/2006), with the primary goal of identifying the aetiological agents of viral AGE outbreaks that were referred to the reference centre for analysis. RVA infection was determined by enzyme immunoassay [EIA, Premier Rotaclone®, Meridian Bioscience, Inc. (Cincinnati, Ohio, USA); Ridascreen®, R-Biopharm (Darmstadt, Hesse, Germany)] according to the manufacturer’s protocol. For all other viruses, viral nucleic acids were extracted from 10% (v/v) faecal suspensions in Tris-Ca^2+^ buffer (pH 7.2), using the QIAmp® viral RNA Mini kit (QIAGEN®, Valencia, CA, USA). Extracted nucleic acids were analysed for the presence of NoV, HAstV, HAdV, HBoV and AiV using conventional and qualitative polymerase chain reaction (PCR), as described previously (Yamashita et al. 2000, Allard et al. 2001, Kapoor et al. 2010, Ferreira et al. 2012). Quantitative PCR (TaqMan® real-time PCR, qPCR) targeting the conserved region of the HAdV hexon (Hernroth et al. 2002) was also performed. HAdV PCR amplicons (301 bp) were purified using the QIAmp^®^ PCR Purification Kit (QIAGEN, CA, USA) and then subjected to sequencing. Both strands of DNA in PCR amplicons were sequenced using an ABI Prism 3100 Genetic Analyzer with the Big Dye Terminator Cycle Sequencing Kit v.3.1 (Applied Biosystem, CA, USA). Centri-Sep columns (Princeton Separations, CA, USA) were used to purify sequencing reaction products prior to analysis in the genetic analyser. A neighbour-joining phylogenetic tree was constructed from the sequences using MEGA 5 software; 1,000 pseudo replicate data sets were used to achieve a Bootstrap value above 70%. The nucleotide sequences reported in this study are listed in the GenBank database under the accession numbers KM099402 - KM099407. Although non-enteric HAdVs are not routinely screened by RRRL-LVCA, nucleotide sequence analysis can be used to identify these viruses in stool samples from patients with diarrhoea.

**TABLE:** Epidemiological and clinical characteristics of the patients analysed for the presence of enteric viruses associated with a gastroenteritis outbreak in Rio de Janeiro, Brazil, 2013

Patient	Age	Gender	Symptoms	Days of diarrhea	PCR for HAdV^b^	Type	qPCR HAdV (Virus concentration)^c^
1^a^	6 months	Male	Fever/diarrhea	03/04	HAdV	A12	2.2 X 10^7^/ 2.8 X 10^7^
2	8 months	Male	Fever/diarrhea	04	HAdV	A12	1.1 X 10^7^
3	18 months	Male	Diarrhea	03	HAdV	F41	1.4 X 10^3^
4	22 months	Male	Fever/diarrhea	04	HAdV	A12	4.2 X 10^6^
5	23 months	Female	Diarrhea	03	ND	ND	ND
6	25 months	Female	Fever/vomit/diarrhea	03	HAdV	A12	3.2 X 10^7^
7	30 months	Male	Fever/diarrhea	04	ND	ND	ND
8	31 years	Male	Diarrhea	03	ND	ND	ND
9	53 years	Female	Diarrhea	04	ND	ND	ND

^a^patient 1: two samples were collected on differents days;^b^all samples were tested for rotavirus (RVA), norovirus (NoV), human adenovirus (HAdV), human astrovirus (HAstV), human bocavirus (HBoV) and Aichi virus (AiV); ^c^DNA copies/g stool, PCR = qPCR+; ND = not detected.

All 10 faecal samples were negative for RVA, NoV, HAstV, AiV and HBoV. Six (60%) faecal samples from five patients (including two samples from patient 1) were positive for HAdV by PCR. HAdV-positive individuals (four male and one female) ranged in age from six-25 months (mean age 16 months). Clinical symptoms included fever (4/5), diarrhoea (5/5), and vomiting (1/5). No viruses were detected in any of the adult patients or in one child, in whom diarrhoea was the sole clinical manifestation, as presented in the Table. Unfortunately, no information about the children’s RVA vaccination status was available.

Viral loads of HAdV A12-positive samples, as determined by qPCR, ranged from 4.2 X 10^6^ to 3.2 X 10^7^ DNA copies/g stool (mean 1.9 X 10^7^). HAdV A12-positive samples had greater viral loads than F41 (1.4 X 10^3^ DNA copies/g stool) (Table).

HAdV sequences were subjected to genetic characterisation, as well as relationship assessment determined by comparisons of nucleotide sequences both among the sequences obtained in this study and the available reference strains in the GenBank database. Five of the six HAdV-positive samples were characterised as non-enteric HAdV A12, with 100% nucleotide identity among these sequences and others from Japan (AB330093.1), India (HQ268779.1 and HQ268780.1) and Gabon (KJ425133.1).

One sequence was characterised as enteric HAdV F41, which is highly similar (98.6% to 100% nucleotide identity) to strains from Japan (AB728839.1), Brazil (JN654703.1, JN654705.1 and JN654706.1) and the USA (KF303069.1, KF303070.1 and KF303071.1) (Figure).

**Figure:**
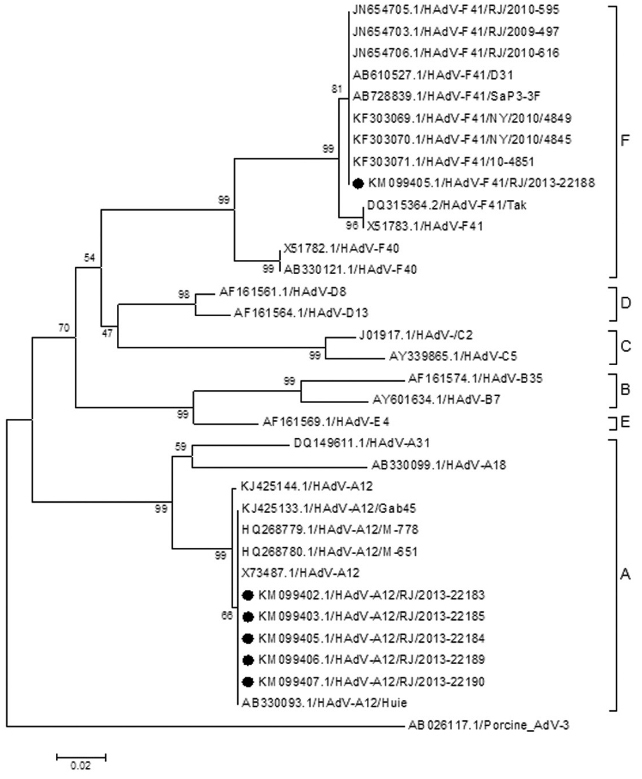
Phylogenetic analysis of human adenovirus based on a partial nucleotide sequence (301 bp) of the Hexon region of the structural gene. Neighbour-joining dendrograms were constructed with the MEGA 5 program. The studied samples are indicated, as well as the GenBank accession numbers and countries of origin. Bootstrap values are above 70%, as estimated with 1,000 pseudoreplicate data sets at each node.

HAdVs were the sole aetiological agents detected in an AGE outbreak in a low-income community in Rio de Janeiro. The non-enteric HAdV A12 was for the first time associated with an AGE outbreak in Brazil. Detection of this type of adenovirus has been reported previously in children with diarrhoea (Ren et al. 2013, Lekana-Douki et al. 2015), including cases of outbreaks (Chitambar et al. 2012), but HAdV 12 has not been associated with AGE until now. The viral loads of the HAdV 12-positive individuals in this study suggest viral replication in the gut. Furthermore, high HAdV viral loads are associated with the high transmissibility of adenoviral diseases. A previous report implicated HAdV A12 as the aetiological agent of AGE outbreaks (Akihara et al. 2005).

Other than RVA, adenoviruses are considered the most important causative agents of gastroenteritis in children under 5 years of age (Elhag et al. 2013), especially enteric HAdV F40/41 (Houpt E, Personal Communication). Enteric HAdV F41 was detected in only one child in this study, who presented a low viral load, possibly due to a previous infection with HAdV F41 because prolonged shedding after HAdV infection has been reported (Akihara et al. 2005).

HAdV A12 Brazilian strains showed homology (100%) with strains obtained from patients with keratoconjuctivitis in Japan and from patients with AGE in India and Gabon (Ishiko et al. 2008, Chitambar et al. 2012, Lekana-Douki et al. 2015). Such shared homology suggests that this strain was imported recently to Brazil. The main symptoms observed in this outbreak were diarrhoea and fever; the child with a higher viral load exhibited more severe disease associated with vomiting. HAdV is associated with moderate to severe AGE, of which the predominant symptoms include diarrhoea, fever, vomiting and abdominal pain (Corcoran et al. 2014).

In conclusion, this study emphasises the importance of identifying unique or rare aetiologic agents associated with AGE. Studies focusing on the prevalence of distinct AGE agents are especially relevant in countries where RVA vaccination is routinely performed, such as Brazil.
